# Supplementary material for: Transcriptomic Insights on the Preventive Action of Apple (cv Granny Smith) Skin Wounding on Superficial Scald Development
Source: Int J Mol Sci. 2021 Dec 14;22(24):13425. doi: 10.3390/ijms222413425 (PMC8705499; doi:10.3390/ijms222413425)
Supplement: Supplementary file 1 [file ijms-22-13425-s001.zip › Supp Table S1.pdf]

Supplementary Table S1

vintage 2020	mean starch content	mean fresh weight	sugars brix°	firmness kg/cm2	acidity g/L mal.ac
harvest	2.0	189	10	8.1	7.9
1 month	*	173	10.8	8.1	8.2
6 weeks	*	140	10	7.6	7.6
3 months	*	164	10.9	6.2	6
6 months	*	161	10.6	6.0	6
