# Supplementary material for: Transcriptomic Insights on the Preventive Action of Apple (cv Granny Smith) Skin Wounding on Superficial Scald Development
Source: Int J Mol Sci. 2021 Dec 14;22(24):13425. doi: 10.3390/ijms222413425 (PMC8705499; doi:10.3390/ijms222413425)
Supplement: Supplementary file 1 [file ijms-22-13425-s001.zip › Supp Table S2.pdf]

Supplementary Table S2

vintage 2019	mean starch content		mean fresh weight		sugars brix°		firmness kg/cm2		acidity g/L mal.ac	
	CTR	WOUND	CTR	WOUND	CTR	WOUND	CTR	WOUND	CTR	WOUND
harvest	2.1	-	213	-	9.7	-	8.4	-	9	-
20 days	2.4	2.4	207	204	11	11	7.74	8.1	9.2	9.3
30 days	2.7	2.7	188	206	10.9	10.7	8.33	7.86	9.1	8.9
90 days	*	*	198	188	12.5	12.5	7.43	7.38	7.9	7.5
