# Supplementary figures and images for: Transcriptomic Insights on the Preventive Action of Apple (cv Granny Smith) Skin Wounding on Superficial Scald Development
Source: Int J Mol Sci. 2021 Dec 14;22(24):13425. doi: 10.3390/ijms222413425 (PMC8705499; doi:10.3390/ijms222413425)

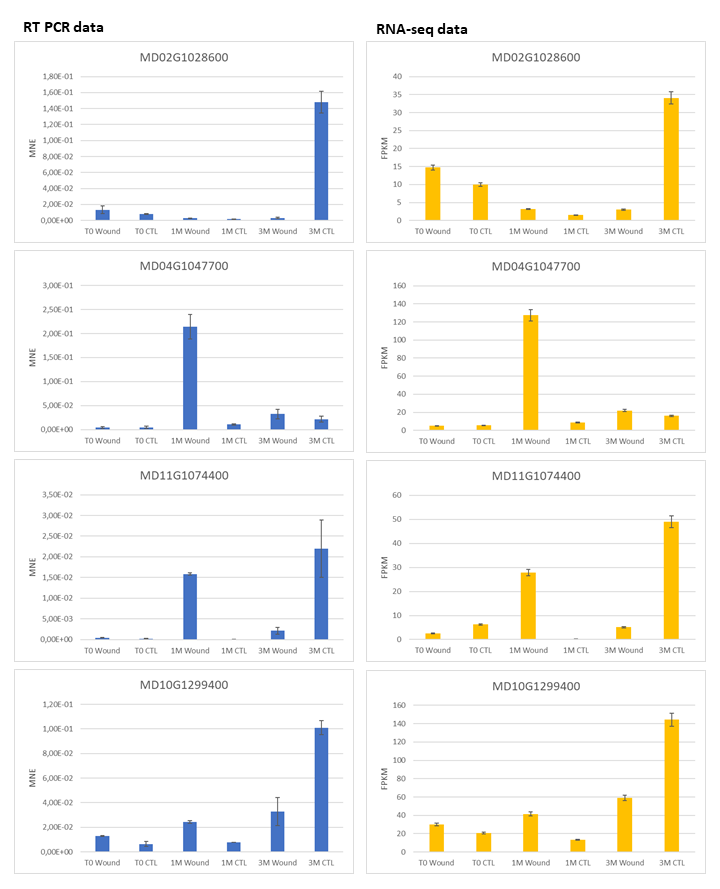

Supplement: Supplementary file 1 [file ijms-22-13425-s001.zip › Supplementary Figure S1.1 - RNA-Seq validatioin.png]

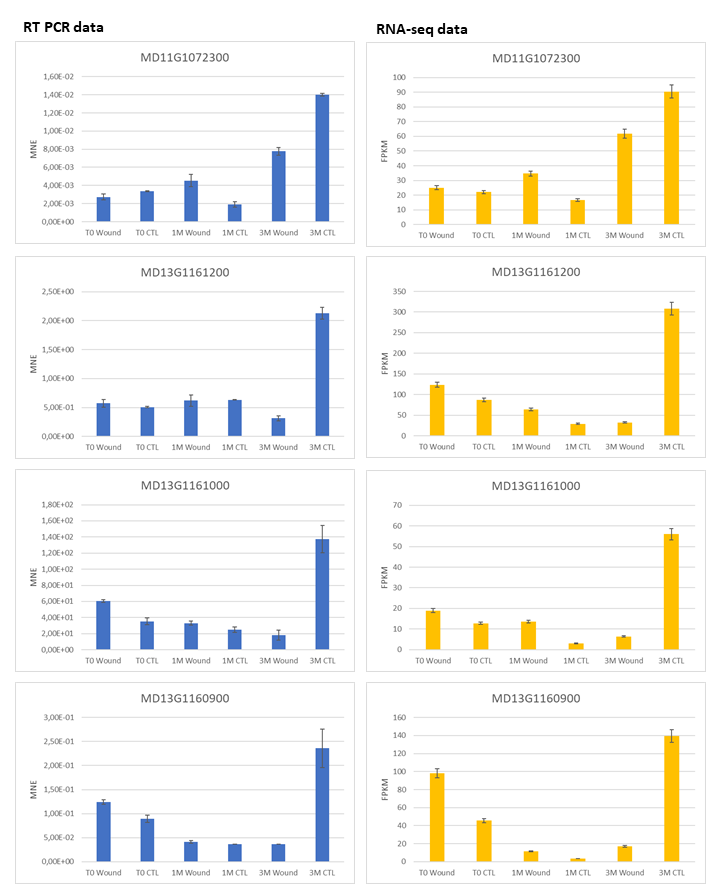

Supplement: Supplementary file 1 [file ijms-22-13425-s001.zip › Supplementary Figure S1.2 - RNA-Seq validatioin.png]

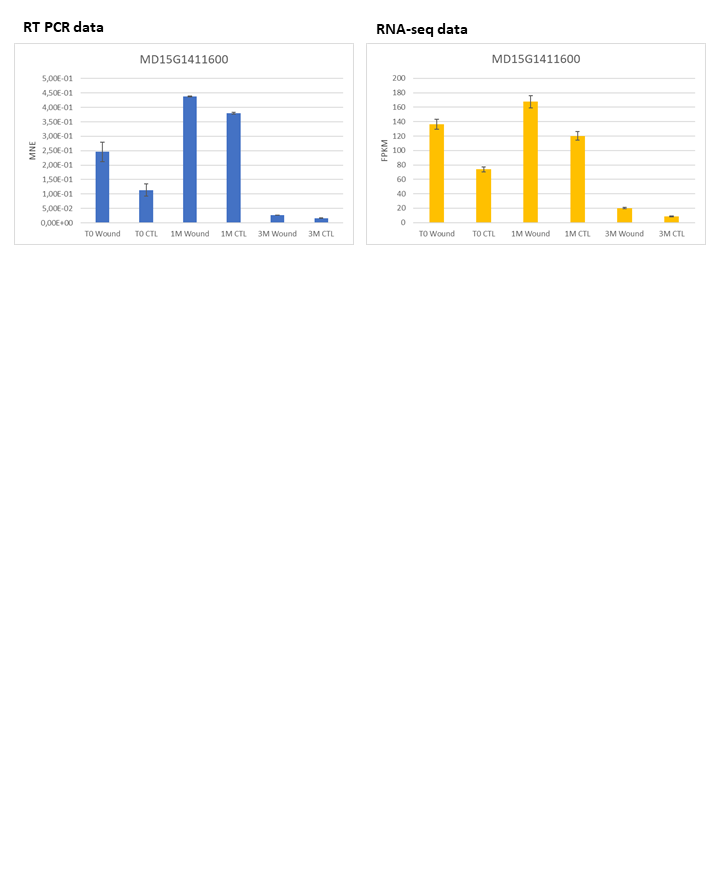

Supplement: Supplementary file 1 [file ijms-22-13425-s001.zip › Supplementary Figure S1.3 - RNA-Seq validatioin.png]
